# Endodontic regeneration: hard shell, soft core

**DOI:** 10.1007/s10266-020-00573-1

**Published:** 2020-12-02

**Authors:** Matthias Widbiller, Gottfried Schmalz

**Affiliations:** 1 Department of Conservative Dentistry and Periodontology, University Hospital Regensburg, Franz-Josef-Strauß-Allee 11, D-93053 Regensburg, Germany; 2 Department of Periodontology, University of Bern, Freiburgstrasse 7, Bern, CH-3010 Switzerland

**Keywords:** Dentin, Pulp, Regeneration, Tissue engineering, Regenerative medicine

## Abstract

A loss of organs or the destruction of tissue leaves wounds to which organisms and living things react differently. Their response depends on the extent of damage, the functional impairment and the biological potential of the organism. Some can completely regenerate lost body parts or tissues, whereas others react by forming scars in the sense of a tissue repair. Overall, the regenerative capacities of the human body are limited and only a few tissues are fully restored when injured. Dental tissues may suffer severe damage due to various influences such as caries or trauma; however, dental care aims at preserving unharmed structures and, thus, the functionality of the teeth. The dentin–pulp complex, a vital compound tissue that is enclosed by enamel, holds many important functions and is particularly worth protecting. It reacts physiologically to deleterious impacts with an interplay of regenerative and reparative processes to ensure its functionality and facilitate healing. While there were initially no biological treatment options available for the irreversible destruction of dentin or pulp, many promising approaches for endodontic regeneration based on the principles of tissue engineering have been developed in recent years. This review describes the regenerative and reparative processes of the dentin–pulp complex as well as the morphological criteria of possible healing results. Furthermore, it summarizes the current knowledge on tissue engineering of dentin and pulp, and potential future developments in this thriving field.

## Introduction

**Fig. 1:**
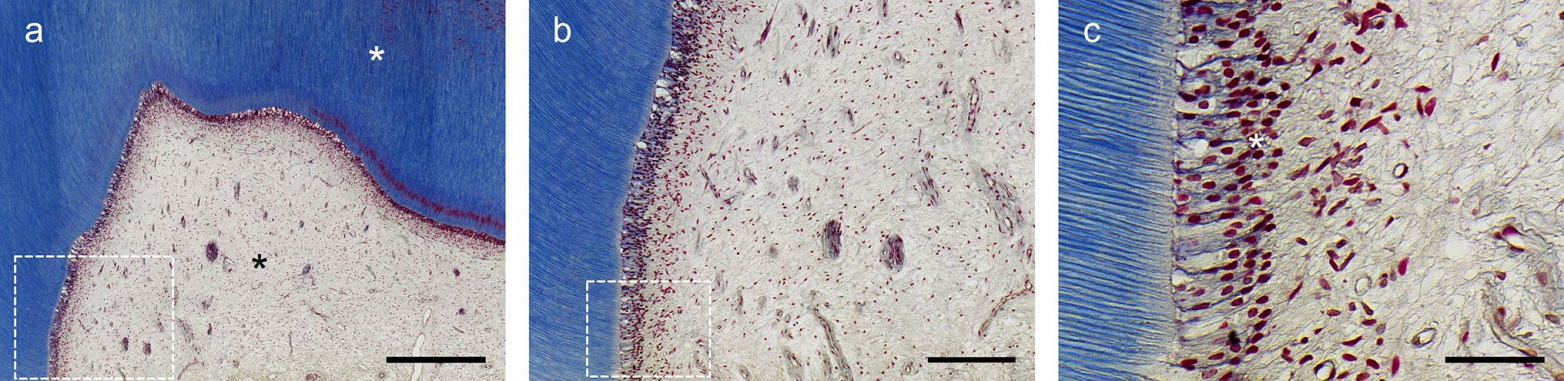
Masson trichrome staining of a healthy molar. **a** Dentine (white asterisk) and pulp (black asterisk) form a closely interconnected tissue complex. **b** Physiologically, dentin has a uniform structure of slightly curved tubules and odontoblasts are located at the interface of pulp and dentin. The pulp core consists of a structured and inflammation-free connective tissue with vascular and nerve components. **c** The primary odontoblasts are of columnar shape and polarized with nuclei located at their basal end (white asterisk). They are arranged in a palisade-like formation and extend their processes into the dentin tubules. Dashed boxes mark the view of the subsequent image. Scale bars: 600 µm (**a**), 200 µm (**b**), 50 µm (**c**)

For healthy and vital teeth, the hard dentin shell provides a strong physical framework for the soft pulp core inside. Both tissues, dentin and pulp, have to be understood as a physiological unity. Anatomically and functionally closely interlinked, the dentin–pulp complex counters external impacts and reacts sensitively to all kinds of stimuli, e.g., caries or trauma. At the pulp chamber, odontoblasts are lined up as single-cell layer and extend their processes wide into the dentin tubules (Fig. 1). Adjacent to the odontoblast layer is the pulp connective tissue, which is permeated by blood vessels, lymphatics and nerve fibers. In addition to fibroblasts, the pulp contains mesenchymal stem cells and tissue-resident immune cells [1, 2]. Via odontoblasts, the pulp tissue is connected to the protective dentin in a zipper-like manner and fulfills several important functions. Besides maintaining the tissue’s metabolic processes, the pulp and, especially, the odontoblasts form tubular dentin during tooth development and the phase of wear, which is described as primary dentin and secondary dentin respectively. In addition, the pulp repels external stimuli through tertiary dentin formation and an immune response [3]. Ultimately it is also a sensory organ that senses changes, e.g., in temperature or pathogenic stimuli [1, 4]. Regeneration as well as repair processes are constantly in progress, however, if the damage is so severe that dentin or even pulp tissue is lost, the desire for tissue regeneration is great. In the following, regenerative aspects in the context of physiological reactions of the whole dentin–pulp complex as well as innovative developments in the field of dentin and pulp regeneration will be discussed.

## Biological principles of regeneration

From a biological point of view, the term regeneration describes a process where organisms aim at replacing or restoring missing body parts or tissues [5, 6]. Accordingly, the starting point of regenerative processes is a wound or tissue loss; however, the path that leads to the restoration of tissues can differ. Whereas some organisms are capable of replacing lost tissues or organs by re-growing them on basis of the remaining tissue (e.g., limbs of amphibians or fins of bony fishes), others are limited to healing without replacement. Interestingly, also wound healing and re-organization after loss of a tissue can be seen as a type of structural regeneration (e.g., skin cuts or bone fractures) [5, 6]. Furthermore, regeneration can not only evolve from original tissue remnants but also occur by conversion of extraneous tissues, which is known as metaplasia (e.g., eye lens of the newt can differentiate from the iris epithelium) [7]. In contrast, some organisms do not make the attempt to replace a lost structure by regeneration but try to compensate for it by growth of the residual tissue or organ, also known as compensatory hypertrophy (e.g., human liver). In some cases, a new tissue is formed that is not an exact copy of the original but sufficiently functional from a physiological point of view. This is called atypical regeneration and describes the formation of an incomplete tissue that may differ in detail from the original, e.g., in shape, size or structure (e.g., regeneration of a lost lizard tail as cartilage tube without vertebral segments and of diminished size) [5, 6]. Eventually, this demonstrates that regeneration is primarily aimed at restoring lost functions rather than simply replacing structures.

**Fig. 2:**
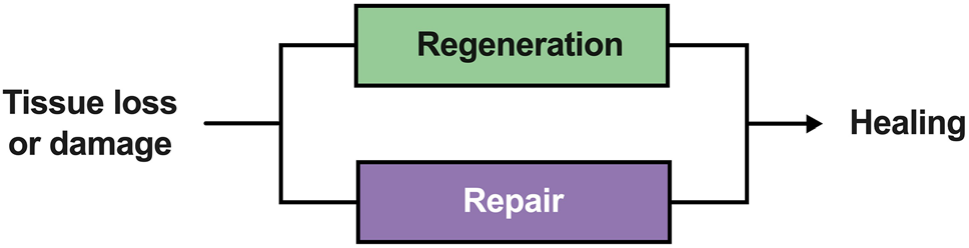
Injured or lost tissue can heal by tissue regeneration or tissue repair. While regeneration restores the tissue to its original state, repair largely creates replacement tissue that substantially differs anatomically or functionally from the original

Especially in the field of dentistry, a non-regenerative healing is often described by the term tissue repair, where newly formed tissue provides structural abnormalities and functional limitations [8, 9]. Both pure regeneration and repair constitute the endpoint of a healing process (Fig. 2); however, repair results in a replacement tissue (scarring) that may also feature regenerative components to a variable extent [8, 10, 11].

Interestingly, a pure tissue regeneration is reported to be highly limited in higher organisms such as mammals, which are likely to form only scar tissue in the process of healing, since this is of only minor relevance for survival [5, 6]. Whereas the human body is known to reestablish for example hepatic tissue or a fingertip after amputation, a similar form of autonomous regeneration of dental tissues does typically not occur. After the decay of enamel and dentin or the loss of the pulp, tissues do not regrow but have to be replaced by synthetic materials. However, thinking about the manifold aspects of regeneration, many dental procedures that are part of everyday dentistry already represent regenerative processes, as will be described in more detail later. In addition, the field of regenerative medicine is an integral part of dental science today and vice versa. In recent years, many ideas from the field of tissue engineering have come to light seeking to restore the dentin–pulp complex in particular.

## Regenerative medicine and tissue engineering

**Fig. 3:**
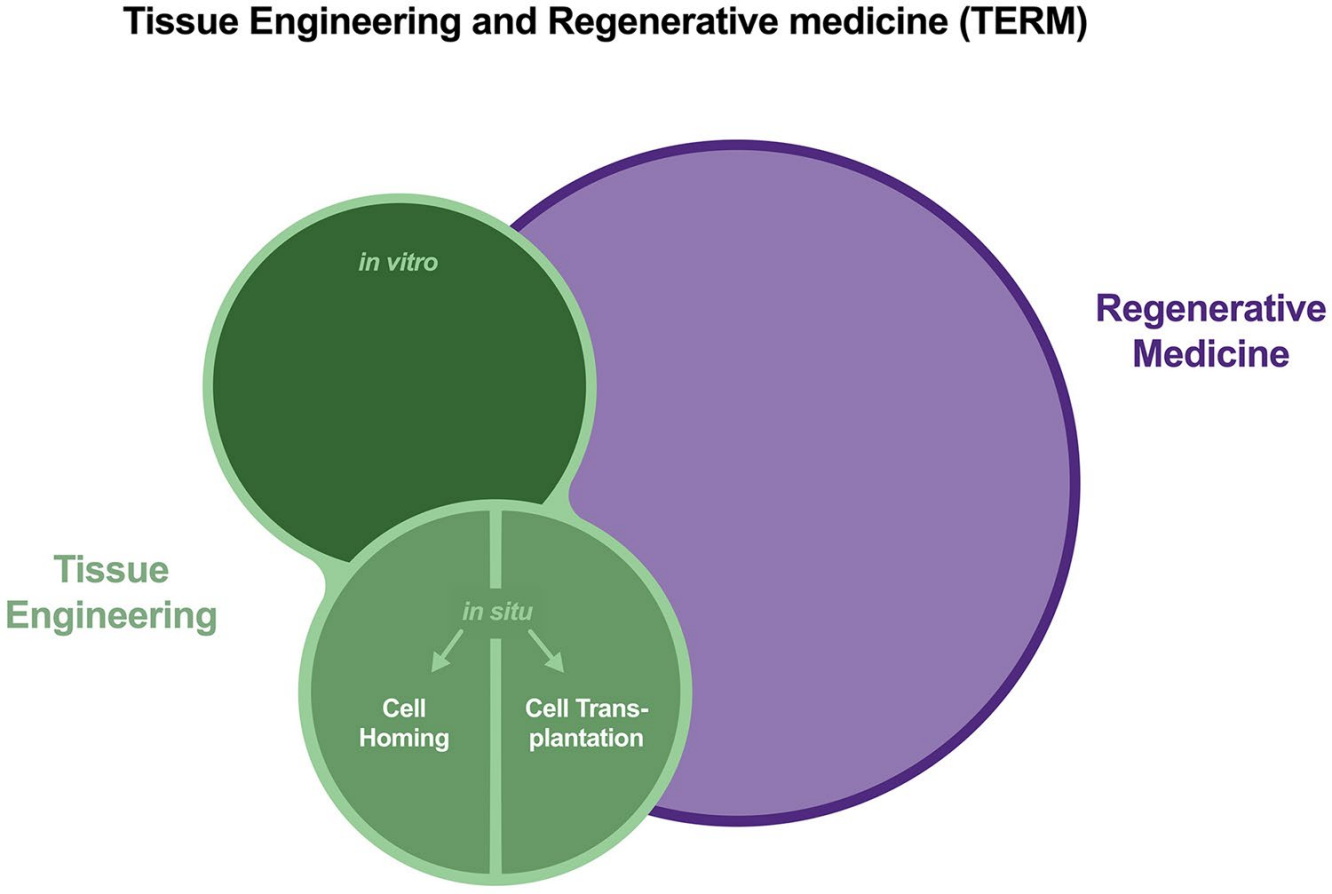
A comparative presentation of the relationship between regenerative medicine (RM) and tissue engineering (TE). Although RM is a broader and more general field than TE, one does not fully encompass the other. Both aim to restore the function of lost or destroyed tissue and can be seen as one research unit: tissue engineering and regenerative medicine (TERM). In the field of TE, a distinction is made between in vitro and in vivo concepts. While functional organs or tissues are constructed and implanted in the first, functionalization takes place in the body in the second approach. For this purpose, cells can be transplanted (cell transplantation), or a suitable scaffold can be used to attract cells from local sources (cell homing)

As defined by Mason and Dunnill, the subject of regenerative medicine aims at the replacement and regeneration of human cells, tissues or organs [12]. The translational branch of regenerative medicine stretches its umbrella over various topics such as gene therapy, stem cell transplantation, use of soluble molecules, cell reprogramming and engineering of tissues [12]. However, tissue engineering can be regarded as a specialty of its own that applies engineering principles based on cells, scaffolds and bioactive molecules to restore tissues or organs [13]. Nevertheless, a clear demarcation of both subjects is not possible as regenerative medicine emphasizes the cellular aspect and the specialty of tissue engineering puts a focus on manufacturing and engineering of tissues (Fig. 3) [14]. The fact that both fields can be seen integrative with regard to their objectives and methodical facets led to the overarching designation as tissue engineering and regenerative medicine (TERM) [15].

In terms of tissue engineering, various concepts have been developed over time that are relevant to medicine and dentistry in particular. On the one hand, there is in vitro tissue engineering, where traditionally scaffolds, cells and bioactive molecules are assembled outside the body and implanted into the body as a functional tissue (Fig. 3) [16]. On the other hand, there is the innovative idea of in situ tissue engineering, which takes place at the site of injury and exploits the human body’s resources, e.g., local cells or bioactive molecules, to rebuild tissues [17, 18]. The implanted structures are primarily not functional but provide the microenvironment to be populated by cells in situ and restore the lost tissue [16].

Seeing the regenerative potential of the human body and the promising concepts of tissue engineering and regenerative medicine, researchers have been seeking to restore teeth over decades [19]. A particular challenge in terms of endodontic regeneration is certainly the fact that the dentin–pulp complex is an anatomically and functionally interconnected tissue. Whereas a full regeneration of the dental pulp from local stem cells seems feasible [20, 21], the regeneration of an acellular hard tissue like dentin using TERM strategies poses a desirable goal for scientists [22]. Though a full regeneration of the entire dentin–pulp complex, which comprises restoration of both components, is hardly conceivable at the moment, many regenerative aspects are already now—sometimes maybe unnoticed—implemented in daily dental practice. Further, in-depth understanding of disease mechanisms of the dentin–pulp complex and its biological potential will certainly change methods and create new possibilities for its preservation and regeneration in the future.

## Regeneration and repair in the dentin–pulp complex

The need for regeneration naturally depends on the extent of tissue destruction or loss. In the same way that anatomically and functionally no clear separation of pulp and dentin is possible, the destruction of both tissues, e.g., by advancing caries or sudden trauma, is a disease of both. However, the historical and pragmatic distinction between reversible and irreversible inflammation of the pulp and irrecoverable destruction of dentin does not go far enough [23, 24]. Rather, it must be recognized that the disease of the dentin–pulp complex is a dynamic spectrum with many biological and spatiotemporal intermediates between the extremes. Accordingly, a consideration of regenerative processes cannot be done in isolation, but in combination of both tissues.

### Dentin

#### Regeneration and repair

**Fig. 4:**
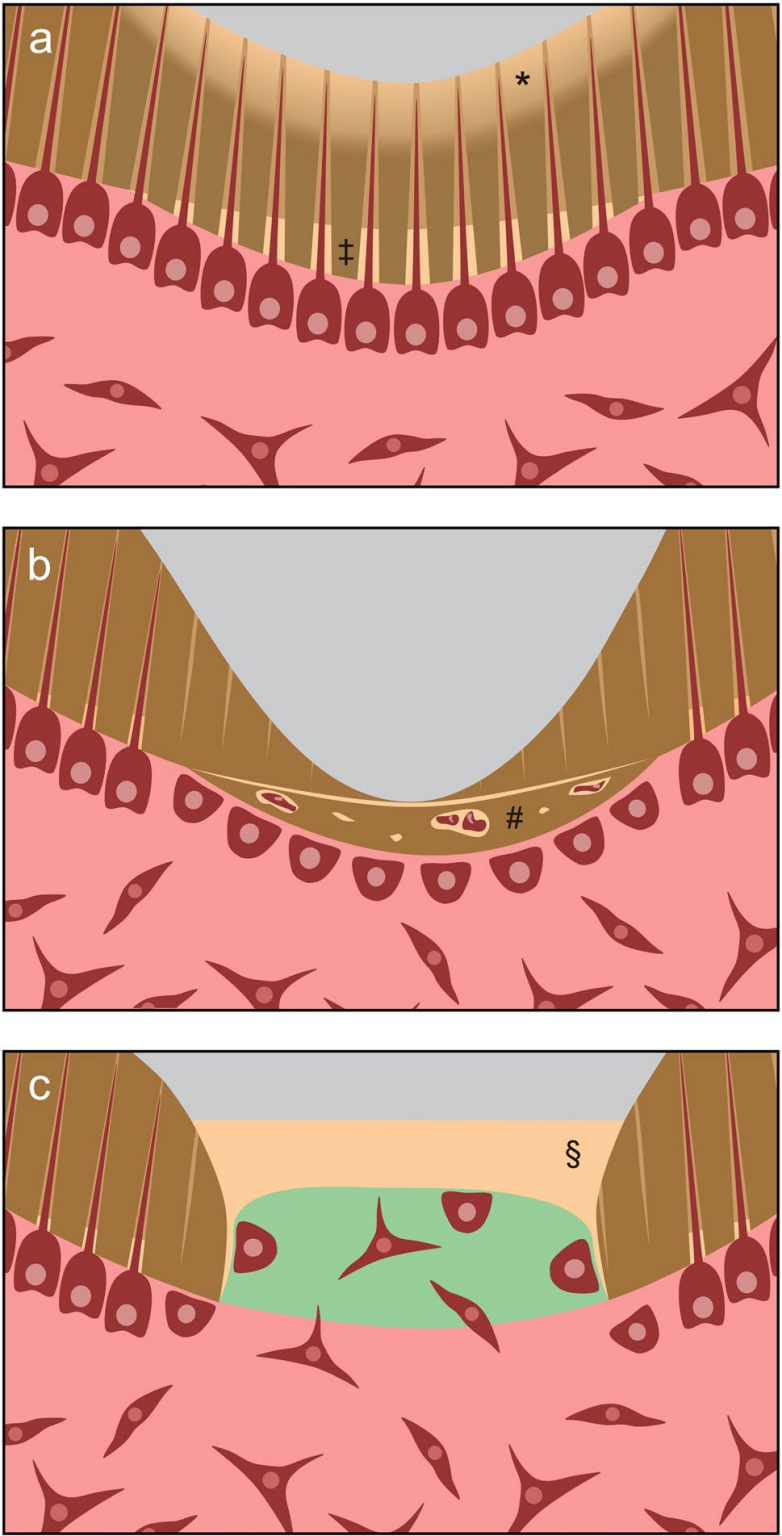
Regeneration and repair processes in dentin. **a** Mechanical preparation, dental materials or caries represent an irritation to odontoblasts that leads to intratubular deposition of minerals as well as reactionary dentin formation (indicated by ‡). Dentine areas demineralized by caries (indicated by *) can be remineralized to a certain extent. **b** Severe stimuli lead to the loss of odontoblasts. The odontoblast-like replacement cells secrete unstructured repair dentin (indicated by #). **c** A tissue engineering approach to dentin regeneration is based on the idea that cells of the pulp migrate into a scaffold (green), form hard tissue (indicated by §) and, thus, seal the dentin wound. In contrast to reactionary or reparative dentinogenesis, there is no narrowing of the pulp cavity

The caries-related decay of dental hard tissue by acid-forming microorganisms has long been regarded as an irreversible and, above a certain level, not self-limiting process [25]. To prevent the infection of the pulp, treatment priority was to remove soft and discolored dentin and subsequently restore the tooth [26, 27]. Over the last years, new concepts of caries removal have been developed and integrated into the clinical treatment spectrum [28, 29]. At asymptomatic teeth or such with reversible pulpitis, deep caries is nowadays incompletely removed to avoid trauma or even exposure of the pulp, which reportedly has a negative effect on the preservation of pulp vitality [30, 31]. Incomplete caries removal can be performed in one or two steps. While in a two-step procedure, softened dentin is left untouched near the pulp and removed a few months later, infected dentin remains behind in pulp proximity without a re-entry in the one-step approach [32]. However, the restorative sealing inactivates the caries lesion due to a shift in bacteria metabolism and cutting off bacterial nutrients, and facilitates remineralization of the softened tissue (Fig. 4a) [28, 33]. Remineralization of affected dentin can be achieved by minerals delivered by pulpal fluids and by lining with ion-releasing dental materials [33, 34].

From the perspective of regenerative biology, the destruction of enamel and dentin by caries is so far considered to be irreparable and cannot presently be regenerated but only substituted by restorative procedures. Nevertheless, affected, pulp-near dentin, which is remineralized after selective caries removal, undergoes some sort of tissue regeneration process that facilitates healing of the dentin–pulp complex without essential structural or functional impairments (Table 1).

**Table 1:** Structural and anatomical criteria for regenerative or reparative processes in the dentin–pulp complex

	Regeneration	Repair
Dentin	Remineralization Reactionary dentin	Reparative dentin Ectopic tissue (bone, cementum, etc.)
Pulp	Intact odontoblast layer Inflammation-free connective tissue	Odontoblast-like cells Pulp stones

Despite dentin contains no cell bodies, it is important to acknowledge it as a vital tissue, which is crossed by odontoblast processes and, thus, physiologically connected to the pulp. External stimuli (e.g., mechanical preparation, dental material components, microorganisms) are perceived by the odontoblast processes and lead to a physiological reaction [35]. Chemicals as well as bacteria and their byproducts can penetrate through the dentin tubules, the number of which increases with proximity to the pulp [36]. Dentin close to the pulp is, thus, very permeable and the barrier function of thin dentin layers is quite low [37]. Odontoblasts react to mild stimuli first by intratubular deposition of minerals and finally by formation of reactionary dentin (tertiary dentin), which reveals tubules in continuity with secondary dentin, however, sometimes sparse and irregularly oriented (Fig. 4a) [2, 3, 38]. Furthermore, not only caries metabolites but also bioactive dental materials like calcium hydroxide or tricalcium silicate cements, which are applied to pulp-near dentin (indirect pulp capping), can promote reactionary dentinogenesis [2, 3]. As this type of dentin forms in a physiological reaction to damage and fulfills both the functional as well as the microanatomical criteria, this reaction can be seen as regenerative process to maintain the health of the dentin–pulp complex (Table 1).

However, if the pulp is encountered by more intense stimuli, e.g., in the case of rapidly progressing deep caries, or exposed in the course of excavation, its regenerative potential may be exhausted and other mechanisms come into effect [38]. In this situation, the odontoblast layer perishes locally or gets disrupted, so that no primary odontoblasts are left to augment the affected residual dentin layer or to close the pulp cavity again [3, 39, 40]. If the inflammatory state of the pulp permits, stem cells are now activated and migrate to the site of action, where they differentiate into odontoblast-like cells and secrete hard tissue (Fig. 4b). The formed dentin is now named reparative dentin (tertiary dentin), because, unlike reactionary dentin, it has an irregular, mostly atubular structure with occasional cell inclusions [3, 40]. When bioactive materials are applied directly onto exposed or amputated pulp tissue (direct pulp capping, pulpotomy), reparative dentin is deposited, which is generally referred to as bridging [41–43]. From a regenerative-biological point of view, this process can be seen as a repair of the pulp wound, which aims at healing of the dentin–pulp complex, however, without complete restoration of the tissue’s original microanatomy (Table 1). Finally, if the microbiological load is too heavy and the pulp inflammation becomes uncontrolled and destructive, the tissue is at risk of necrosis and can neither form reactionary nor reparative dentin.

In summary, it is apparent that many routine activities in everyday dental care are already based on physiological regeneration and repair mechanisms, depending on whether the dentin–pulp complex is challenged by slight (e.g., selective caries removal, indirect pulp capping) or more intense stimuli (e.g., direct pulp capping or pulp amputation).

#### Tissue engineering

At the same time, there are interesting attempts to regenerate dentin according to the principles of tissue engineering [22]. In contrast to the replacement of destroyed dentin by bioactive materials, which induces the physiological reaction of the pulp leading to a narrowing of the pulp space (Fig. 4a,b), the idea behind dentin regeneration is to replace lost dentin at the site of damage and repair the dentin–pulp complex (Fig. 4c).

In this context, the research group around Paul T. Sharpe reported on a translational tissue engineering approach to replace lost hard tissue by naturally formed reparative dentin secreted by mobilized pulp stem cells [44]. In terms of in situ tissue engineering, a biodegradable collagen matrix was used as scaffold and small-molecule GSK-3 inhibitors, which are connected to reparative dentinogenesis and act as Wnt agonists [45], served as signaling molecules [44]. After application onto the exposed pulp of mice, the scaffold was colonized by pulp cells and almost complete mineralization and closure of the lesion occurred (Fig. 4c) [44]. In a follow-up study, the authors took a closer look at the hard tissue formed in this model, now also in rats with even larger defects [46]. The formed tissue resembled dentin in its mineral composition, but no tubular structure was observed. Furthermore, it was shown that the pharmaceutical activity of the small-molecule inhibitor was spatially limited and without any systemic side effects [46].

In the light of regenerative biology, it is clear that this is a tissue replacement in the sense of a biological repair since the newly formed dentin does not provide a tubular structure. However, enhancement of reparative dentin formation appears to be translatable into clinical therapy by a direct pulp capping approach. It is of course debatable whether in situ tissue engineering of dentin offers a clinical advantage in view of the small tissue volumes involved, but the investigations definitely reveal interesting aspects and open up a new field of research. Furthermore, signaling molecules can be incorporated not only in tissue engineering scaffolds but also in dental materials to achieve desired biological effects.

### Pulp

#### Regeneration and repair

Regardless of whether dentin is formed in the course of tooth development, in response to stimuli or in tissue engineering processes, the dental pulp is the formative organ and its health is the essential prerequisite. However, the pulp is simultaneously compromised by an infection-related immune response as soon as dentin is reached by microorganisms [47] and can only maintain its defensive functions to a certain degree. Besides reactionary or reparative dentin formation, humoral and cellular immune mechanisms are activated resulting in an initially reversible, local inflammation. A widespread invasion of bacteria leads to irreversible pulpitis, which is characterized by an intense and spatially spreading inflammation [2, 48].

As the most peripheral cells in the dentin–pulp complex, odontoblasts with their long intratubular processes are the first to be encountered by antigens and trigger the innate immune response of the pulp tissue. Further on, dendritic cells position themselves strategically and support the odontoblasts in antigen presentation [2, 3]. Generally, pathogenic stimuli are detected by pattern recognition receptors (PRRs) such as the toll-like receptor family (TLRs). With their help, pathogen-associated molecular patterns (PAMPs) are identified and an immune reaction is initiated. The stimulation of TLRs on odontoblasts induces the secretion of proinflammatory cytokines and antimicrobial peptides, which results in the recruitment of circulating immune cells and an immediate destruction of the invading bacteria [4, 49]. Subsequently, the immune response intensifies with increasing depth of the caries lesion, and T-lymphocytes, B-lymphocytes, neutrophilic granulocytes and macrophages accumulate progressively [2, 3]. Additionally, capillaries start sprouting in an initially circumscribed area of the tissue, and nerve fibers interact with dendritic cells in the course of inflammation [50].

To protect the pulp from collateral damage by degradative molecules and enzymes, which are released by immune cells, it is necessary to limit and control the immune reaction [2, 3]. A requirement for healing is that the inflammation does not spread or intensify further but stagnates and is finally resolved. However, this can only occur if the microbiological infection in the pulp is brought under control primarily through clinical intervention, i.e., (selective) removal of the caries, indirect or direct pulp capping and tight closure of the cavity. The residual microorganisms in the pulp tissue are subsequently targeted by local immune cells. Only when all microorganisms are eliminated and the inflammation is resolved, tissue homeostasis can be restored, and healing can take place. In the course of healing, changes in the tissue that appeared during immune reaction regress and the pulp is reorganized structurally [2, 3]. The only sign that might indicate an attempt of tissue repair in the dental pulp after inflammation has subsided is ectopic calcification, commonly known as pulp stones. Both odontoblasts and pulp fibroblasts may be involved in the random mineralization, forming pulp stones with or without a tubular structure respectively (true or false pulp stones) [51]. Here, pulp stones could be described as a kind of scarring, while the surrounding tissue may recover immaculately (Table 1).

Since inflammatory reactions of the dental pulp are primarily irritation-related and localized, severely affected areas can be selectively removed in the course of therapy. Whereas the pulp tissue as a whole was considered to be either reversibly or irreversibly damaged based on clinical criteria in the past, modern therapy approaches recommend amputation of locally highly inflamed areas based on intraoperative criteria like color and bleeding [24, 47, 52]. The amputation of pulp parts or the entire crown pulp (partial or total pulpotomy) can, thus, facilitate healing of the residual tissue even in cases of supposedly irreversibly damaged conditions [53–55].

From a regenerative-biological perspective, healing of the pulp with an intact odontoblast layer seems to be based on regenerative principles, because the original condition can be completely restored within physiological limits (Table 1). If odontoblasts perish due to injurious stimuli or pulp exposure, they are replaced by odontoblast-like cells, which appear more cubic in shape and secrete reparative dentin [3, 56]. Although formation of reparative dentin enables regenerative healing of the pulp connective tissue, the microanatomy of the dentin–pulp interface differs from the original, so that the overall result must be considered as tissue repair with regard to the entire dentin–pulp complex. However, if the damage is too intensive or too widespread, healing by both regeneration and repair is no longer possible and necrosis of the entire tissue occurs.

#### Tissue engineering

**Fig. 5:**
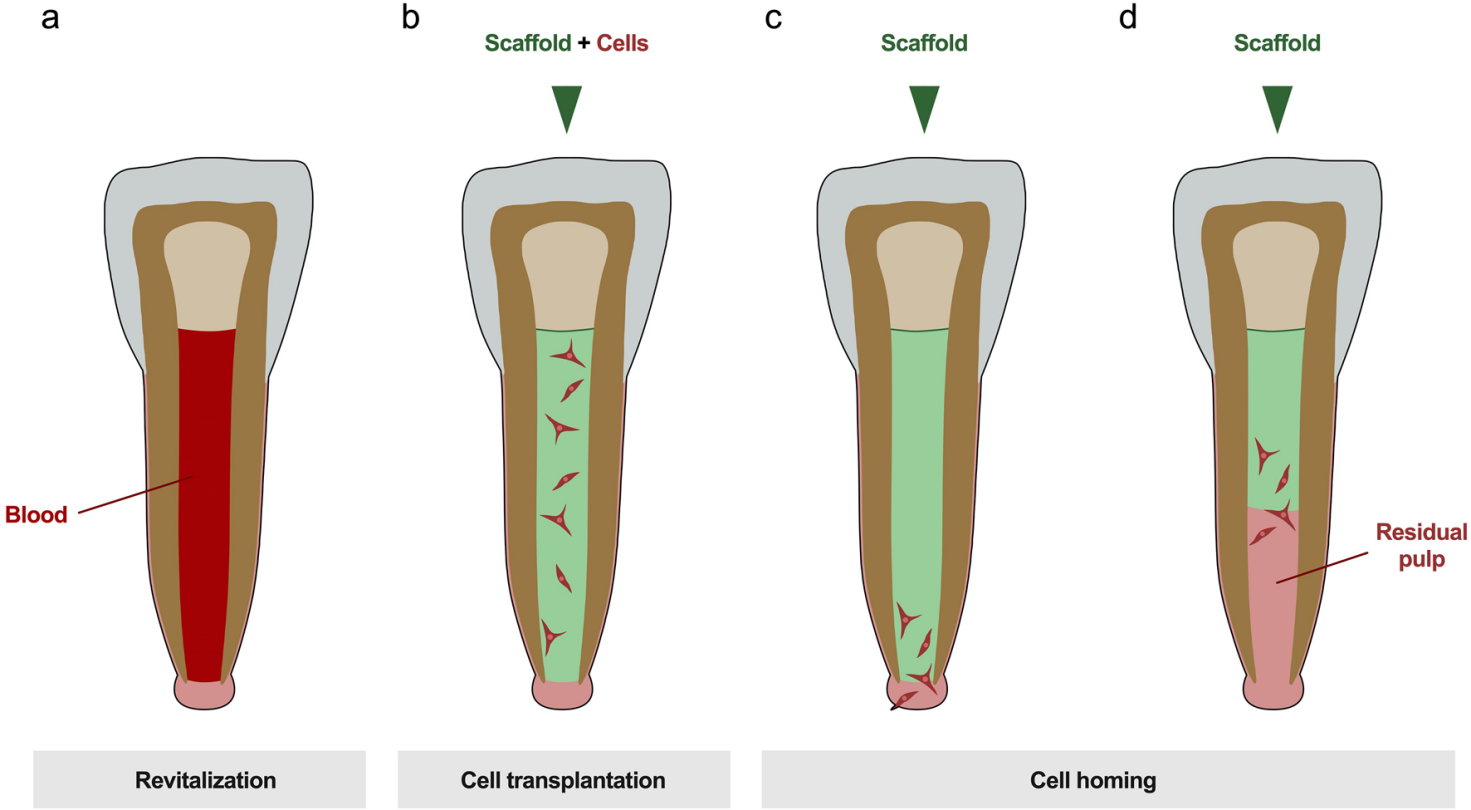
Regenerative endodontic treatment approaches. **a** Revitalization is the process of inducing apical bleeding by which cells are brought into the root canal. **b** In the idea of in situ tissue engineering cells are expanded ex vivo and transplanted into the root canal with a scaffold. **c** Cell homing, on the other hand, is a primarily cell-free approach that makes use of locally available cell sources. A custom-made scaffold with biomolecules is placed in the canal and cells migrate from the periapical tissues. **d** This concept is also applicable if there is residual pulp tissue in the root canal, which in this case serves as cell source and is, thus, expanded

When the pulp tissue falls victim to extensive microbiological infection and becomes severely inflamed or necrotic, it has to be removed and root canal treatment is initiated. During this process, the canals are thoroughly disinfected and filled with a synthetic material. As a result, all functions of the pulp are lost, and the survival of the tooth can be compromised [1, 57]. This has a particularly significant consequence for immature teeth whose root development has not yet been completed. Due to their short roots and thin dentin walls, these teeth are predestined for fractures [58, 59]. A way to regenerate the pulp would allow the root development to be completed and significantly improve the prognosis [60, 61]. This desire initially led to the development of revitalization, a first attempt to regenerate the dental pulp, in the early 2000s [62, 63]. Therefore, bleeding into the root canal is induced and the formed blood coagulum is covered with a bioactive cement (Fig. 5a). In the course of time, a vascularized and innervated tissue forms, which can restore selected functions of the original pulp [21, 64]. Revitalized teeth can develop a sensitivity to cold, periapical inflammation subsides and root development may progress [65, 66]. Histologically, however, this tissue is quite dissimilar to the original pulp. A structured odontoblast layer is typically not observed and ectopic components like cementum or bone are frequently found [67–69].

The result of this treatment approach can, therefore, be regarded as classical tissue repair in a biological sense (Table 1). This is taken into account in the nomenclature of regenerative endodontics as Diogenes et al. terminologically specify this outcome as “guided endodontic repair” [70]. Nevertheless, the clinical results are desirable and promising, and the procedure has become an important part of clinical endodontics today, as the current treatment recommendations of the professional societies indicate [71, 72].

Due to the dissatisfaction with the histological result and the low predictability of the revitalization treatment, researchers made great efforts to achieve pulp regeneration with the help of tissue engineering and regenerative medicine strategies. Many different concepts and ideas have been developed and examined for clinical feasibility over the past years [20, 73]. For in situ regeneration, cell-based procedures have been described as successful, where pulp cells have been expanded ex vivo and inserted into root canal by transplantation in prefabricated scaffolds with stromal-cell-derived factor-1 (SDF-1) [74, 75]. This approach has already been proven in a case series on human participants [76]. Meanwhile, promising results could also be achieved with the implantation of human deciduous pulp stem cells in a controlled clinical trial (Fig. 5b) [77].

Though this is a fascinating approach, its clinical translation confronts scientists and clinicians with considerable challenges. To obtain cells, an additional, healthy tooth needs to be discarded (e.g., a deciduous tooth, permanent teeth to be removed for orthodontic reasons or wisdom teeth in need of removal) or cells would have to be stored in a cell bank beforehand [76, 78].The isolation and expansion of cells requires a lot of time, sophisticated laboratory techniques and is, thus, associated with high costs. Therefore, the concept of in situ tissue engineering has drawn more attention in this field over the last years. The goal is to exploit the body’s regenerating capacity by utilizing endogenous sources of stem or progenitor cells [79, 80]. This bypasses an ex vivo cell manipulation and merely creates a microenvironment for the homing of local cells to regenerate the pulp tissue (Fig. 5c) [17, 18, 81]. In addition to the cells, blood vessels and nerve fibers access the scaffold material and a new tissue is to be formed that corresponds to the original pulp. An important factor here is the selection of the optimal signaling molecules, whereby individual growth factors, combinations of molecules and endogenous proteins from the root canal or blood plasma have been discussed [21, 79, 80, 82]. Furthermore, many studies have been concerned with the questions of optimal scaffold material properties and aimed at developing customized scaffolds for pulp tissue engineering [80, 83–86]. Despite promising results from animal studies and experimental case reports, the translation of this concept into humans is still to come [84, 87, 88].

Interestingly, there are also efforts to regenerate the dental pulp only partially on basis of remaining intact pulp tissue. This would come to fruition if, as described above, irreversibly damaged parts of the pulp are amputated but an intact pulp stump with regenerative capacity is left in the root canal. With the help of in situ tissue engineering lost parts could be restored by mobilizing cells from the residual pulp tissue into a scaffold material and virtually expanding the pulp again (Fig. 5d) [69, 89].

## Conclusion

In many ways, dentin and pulp form a functional and anatomical unit. This applies not only to the pathology of the dentin–pulp complex and its immune reaction, but also to its regenerative or reparative processes that can run concurrently. The initial response to tissue destruction is characterized by regenerative operations, which can—depending on its severity—merge into tissue repair later on. Although the causal and concomitant inflammatory reaction provides the impetus for tertiary dentinogenesis and recruits immune cells on the one hand, its subsidence is the prerequisite for healing.

Innovative approaches to regenerate dentin–pulp complex typically focus on only one tissue, whereby the interrelation of the hard dentin and the soft pulp must be considered anatomically and functionally. Dentin is the product of the pulp and one cannot be seen without the other. A sufficient dentin regeneration can only be based on a physiological pulp tissue and for pulp regeneration, a competent dentin is of great importance. The full and simultaneous regeneration of the entire dentin–pulp complex, however, is currently a far-reaching goal.
